# Association between periodontitis and the risk of palindromic rheumatism: A nationwide, population-based, case-control study

**DOI:** 10.1371/journal.pone.0182284

**Published:** 2017-08-03

**Authors:** Ching-Heng Lin, Der-Yuan Chen, Wen-Cheng Chao, Tsai-Ling Liao, Yi-Ming Chen, Hsin-Hua Chen

**Affiliations:** 1 Department of Medical Research, Taichung Veterans General Hospital, Taichung, Taiwan; 2 Department of Healthcare Management, National Taipei University of Nursing and Health Sciences, Taipei, Taiwan; 3 Division of Allergy, Immunology, and Rheumatology, Department of Internal Medicine, Taichung Veterans General Hospital, Taichung, Taiwan; 4 School of Medicine, National Yang-Ming University, Taipei, Taiwan; 5 Institute of Biomedical Science and Rong-Hsing Research Center for Translational Medicine, Chung-Hsing University, Taichung, Taiwan; 6 School of Medicine, Chung-Shan Medical University, Taichung, Taiwan; 7 Department of Medical Education, Taichung Veterans General Hospital, Taichung, Taiwan; 8 Division of Chest Medicine, Department of Internal Medicine, Taichung Veterans General Hospital, Taichung, Taiwan; 9 Institute of Public Health and Community Medicine Research Center, National Yang-Ming University, Taipei, Taiwan; Katholieke Universiteit Leuven Rega Institute for Medical Research, BELGIUM

## Abstract

**Objective:**

To estimate the association between a history of periodontitis (PD) and the risk of incident palindromic rheumatism (PR).

**Methods:**

Using a nationwide, administrative database, this study identified 4,421 newly-diagnosed PR cases from 2007 to 2012 and randomly selected 44,210 non-PR controls matched (1:10) for sex, age and the year of the index date. After adjusting for comorbid diabetes mellitus, we estimated the odds ratios (ORs) with 95% confidence intervals (CIs) using conditional logistic regression analysis to quantify the association between a history of PD and the risk of PR. The influences of the lag time and severity of PD were examined by calculating ORs for subgroups of patients based on the time interval between the last PD-related visit and the index date and PD-related cumulative cost and number of visit.

**Results:**

This study showed an association between a history of PD and incident PR (OR, 1.51; 95% CI, 1.41–1.61). The association remained significant after variation of PD definitions. The magnitude of the association was greater in those with shorter lag time between the latest date of PD diagnosis and PR index date and those who had a higher number of visits for PD or a greater cumulative cost for PD-related visits. After excluding 569 PR patients who developed rheumatoid arthritis after the index date, we found a consistent time- and dose-dependent association between PD and PR risk.

**Conclusion:**

This study demonstrated a time- and dose-dependent association between PD exposure and PR risk.

## Introduction

Palindromic rheumatism (PR) was first described in 1944 [1]. PR is a clinical syndrome characterized by recurrent episodes of acute arthritis or peri-arthritis with single-joint or multi-joint involvement [1]. PR manifests as pain with or without redness or swelling, lasting from a few hours to days with variable symptom-free periods [1]. In 1992, Guerne and Weismann proposed diagnostic criteria for PR,[2] including (1) a 6-month history of brief sudden-onset and recurrent episodes of monoarthritis or, rarely, polyarthritis or of soft tissue inflammation; (2) direct observation of one attack by a physician; (3) three or more joints affected in one attack observed by a physician; (4) absence of erosions on radiographs and (5) exclusion of other arthritides. The etiology of PR remains unknown. Although genetic risk factors for its development have been identified [3,4], to the best of our knowledge, no environmental risk factors have been reported.

Some PR patients may develop a chronic connective tissue disease, mainly rheumatoid arthritis (RA) [5]. About one to two-thirds of PR patients developed RA during a period of follow-up [6–11]. RA differs from RA as it does not lead to residual joint destruction [1]. Factors associated with the progression to RA in PR patients included genetic factors [12,13], ultrasonographic findings of synovitis and anti-cyclic citrullinated peptide antibodies (ACPA) [7,14]. Although prior studies failed to differentiate PR from RA using clinical and immunologic data [9,10], some genetic background, such as HLA-DRB1 *0803 [3], showed increased susceptibility to PR but not to RA, suggesting that PR is a disease entity distinct from RA.

Periodontitis (PD) is a common inflammatory disease characterized by chronic destructive inflammation of periodontal tissue, with a prevalence of 50% in the United States in those aged 30 years or older [15,16]. PD is induced by the gingival ‘red complex’ bacteria, including *Treponema denticola*, *Tannerella forsythia*, and *Porphyromonas gingivalis (P*. *gingivalis)* [17,18]. In recent years, PD has been identified an environmental risk factor for RA development [19]. PD is linked to RA development through *P*. *gingivalis*, which is the only microorganism that expresses peptidyl-arginine deiminase (PAD) [20], irreversibly converting arginine to citrulline. With the accumulation of citrullinated peptides, immune tolerance to these peptides may be broken, followed by the development of ACPA, which plays a significant role in the pathogenesis of RA [21,22]. Our previous epidemiologic data revealed an association between PD and RA risk, in a dose- and time-dependent manner [23,24]. Another hospital based, case-control study using non-smoking newly diagnosed RA cohort has confirmed this association [25].

Although the association between PD and RA has been rigorously studied in recent years, to the best of our knowledge, no research has investigated the association between PD and PR risk. The aim of this study was to examine the association between a history of PD and the risk of newly diagnosed PR using a longitudinal, nationwide, population-based administrative dataset.

## Methods

### Ethics statement

The Institutional Review Board (IRB) of Taichung Veterans General Hospital (IRB number: CE16251A) approved this study. Because personal information traced was anonymised before data analysis, informed consent was not acquired.

### Study design

The study used a retrospective matched case-control design.

### Data source

The data source was the 2003–2013 claims data from the Taiwanese National Health Insurance Research Database (NHIRD). In 1995, Taiwan started a compulsory National Health Insurance (NHI) program, which currently covers more than 99% of the population in Taiwan. The NHIRD incorporates data regarding dental services, outpatient services, inpatient care, traditional medicine services and drug prescriptions. Therefore, prescription of PR-related medications, including disease-modifying antirheumatic drugs (DMARDs), corticosteroids (CSs), and nonsteroidal anti-inflammatory drugs (NSAIDs), could be identified. Some personal history data, such as smoking, drinking or examination results, were not available in the NHIRD. The Bureau of NHI (BNHI) has regularly conducted random checks of medical charts to improve the accuracy of the NHIRD.[26] The National Health Research Institute processed the database and made it feasible for researchers to use the NHIRD to conduct epidemiologic studies.

This study utilized the 2003–2013 claims data, consisting of enrollment file, inpatient services, and outpatient services, from the NHIRD to identify all newly diagnosed PR patients during 2007–2012. Also, the NHIRD constructed a representative longitudinal health insurance database (LHID2000) of one million people selected randomly from among all enrollees who received services in 2000. The data of the non-PR controls were extracted from the 2003–2013 LHID2000.

The BNHI also has a registry for catastrophic illness patients (RCIP), who have major diseases, such as malignancy and certain connective tissue diseases, including RA, systemic lupus erythematosus, Sjögren’s syndrome (SS), systemic sclerosis (SSc), polymyositis (PM) and dermatomyositis. Only those whose diagnoses were validated by at least two qualified specialists after a thorough review of medical charts were issued a certificate for ‘catastrophic illness’ and were exempt from copayment.

### Definition of PR

PR patients were defined as patients having at least three ambulatory visits or one hospital admission with a diagnosis of PR (International Classification of Diseases, Ninth Revision, Clinical Modification [ICD-9-CM] code 719.3) during 2003–2013. In Taiwan, the criteria proposed in 1992 by Guerne and Weismann [2] were used to diagnose PR.

### Definition of RA

RA patients were defined as individuals who were registered in the RCIP for RA diagnosis (ICD-9-CM code 714.0). The date of enrollment in the RCIP for RA was defined as the index date of RA diagnosis.

### Study subjects

#### PR cases identified from entire population in Taiwan

All newly diagnosed PR patients from 2007 to 2012 were included in the study. Patients who were first diagnosed with PR before 1 January 2007 were excluded. The index date of PR cases was defined as the date of the first outpatient or inpatient visit with a diagnosis of PR. Given a high correlation of RA with PD and PR, we excluded those who had an RA diagnosis before the index date. Because SS, particularly secondary SS, is also associated with both PD [27,28] and PR [29], we excluded patients registered in RCIP with a diagnosis of SS and SS-related diseases, including SLE, SS, SSc, PM or dermatomyositis before the index date.

#### Matched non-PR controls from a representative population of one million

We defined non-PR patients as individuals who had never had an ambulatory or inpatient diagnosis of PR during 2003–2013. From the LHID2000, we randomly selected 10 non-PR controls, using propensity score matching to include baseline differences between cases and controls. The study utilized a multivariable logistic regression model to estimate the propensity score. Sex, age and the year of the index date (index year) were included in the model. We also excluded patients who were registered in RCIP, initially diagnosed before the index date, for RA, SLE, SS, SSc, PM or dermatomyositis. The index date for non-PR controls was defined as the date of the first outpatient or inpatient visit in the index year for any reason.

### Definition of PD

For the improvement of dental health, the BNHI encourages enrollees to undergo routine dental scaling every six months. If individuals do not have PD, the BNHI does not reimburse for more than two dental scalings in a single year. It is possible that patients without PD who received dental scaling at a routine dental check-up could also be coded with a diagnosis of PD (ICD-9-CM codes 523.3–5). For this reason, this study defined PD patients as those having one or more dental visits with a diagnosis of PD and concurrent periodontal or antibiotics treatment or having three or more dental visits with a diagnosis of PD and concomitant dental scaling within one year.

### Proxy measures of PD severity

In this study, we made an assumption that the severity of PD positively correlated with the cumulative number and costs of PD-related visits. To examine the possibility of a dose-response relationship between PD exposure and PR risk, we used the number of visits for PD and the cumulative cost of PD-related visits categorised based on the 25^th^, 50^th^, and 75^th^ percentiles (i.e., quartile [Q]1–Q4) before the index date as proxy measures of PD severity. We summed all expenses of ambulatory visits of patients with a diagnosis of ICD-9-CM codes 523.3–5 before the index date to get the cumulative PD-related cost. The cost was presented in United States dollars (US$), using a conversion rate of 30 new Taiwan dollars (NT$) to 1 US$.

### Interval between latest PD exposure date and the index date

To test the time-dependent relationship between PD and incident PR, we categorised patients with a history of PD into 5 groups according to the time interval between the latest PD-related visit and the index date (with the options <3 months, 3–6 months, 6 months to 1 year, 1 year to 3 years and >3 years).

### A potential confounder

Since diabetes mellitus (DM) is a major risk factor for PD and may be differentially distributed between cases and controls, this study included treated type 1 and type 2 DM (ICD-9-CM codes 250.x) as a potential confounder. Patients were defined as having DM when they had had at least one ambulatory visit that resulted in a diagnosis of DM (ICD-9-CM codes 250.x) with the concurrent prescription of anti-diabetic drugs within one year before the index date.

### Subgroup analyses

We conducted subgroup analyses to determine whether the associations between history of PD using various definitions based on ICD-9-CM and PR were consistent among the subgroups stratified by age, sex, DM, or the status of progression to PR during the entire follow-up period using multivariable analyses.

### Sensitivity analysis

We conducted sensitivity analyses by excluding PR patients who developed RA after the index date and re-selecting non-PR controls matched for age, sex and the year of the index date. RA diagnosis was confirmed if patients were issued a catastrophic illness certificate for RA.

### Correlations of PR-related medication use with PD severity and interval between latest PD exposure date and the index date among PR patients

We further used the data of all PR patients (n = 4,421) to investigate the relationships between PD severity/interval between the latest PD exposure date and the index date and PR-related therapy during the first year of follow-up. We conducted several case-control studies on patients treated with various PR-related medications during the first year as cases and those who did not use corresponding medications during the first year as controls. We quantified the corelations by calculating crude and adjusted ORs with 95% CIs using logistic regression analyses.

### Association between PD and the risk of progression to RA among all PR patients

We used a cohort study design to examine the associations of the risk of progression to RA with PD (using various definitions), PD severity (using the number and cost of PD-related visits as proxies), or the time interval between the latest PD visit and the PR diagnosis among all PR patients (n = 4,421). We quantified the correlations by calculating crude and adjusted hazard ratios (HRs) with 95% CIs using Cox proportional hazard regression analyses.

### Statistical analysis

Continuous variables were presented as a mean ± standard deviation, and categorical variables were presented as a percentage of patients. We tested the differences for continuous variables using Student’s *t*-test and for categorical variables using Pearson’s χ^2^ test. The association between PD and PR risk was quantified by calculating odds ratios (ORs) with 95% confidence intervals (CIs) using conditional logistic regression analysis after adjusting for comorbid DM. We examined the significance of interaction effect by age group, sex or DM on the association between PD and PR risk by estimating the probability (*p*)-value of the coefficient associated with the product of the indicator of age, sex or DM and the indicator of PR using the Wald test. We calculated ORs with 95% CIs using logistic regression analysis after adjusting for age, sex, and comorbid DM to quantify the correlations of PR-related medication use with PD severity and the time interval between the latest PD exposure date and the index date. To determine the association between PD and progression to RA among all PR patients, we calculate crude and adjusted HRs with 95% CIs of progression to RA in patients with a history of PD compared with non-PD patients using Cox proportional hazard regression analysis. A two-tailed *p*-value <0.05 was considered statistically significant. All statistical analyses were performed using SAS statistical software, version 9.3 (SAS Institute, Inc., Cary, NC, USA).

## Results

We identified 4,421 PR cases and randomly selected 44,210 non-PR controls by propensity score matching (1:10) for age, sex and the year of the index date. Table 1 compares baseline demographic and clinical data between PR cases and non-PR controls. The age among PR cases ranged from 6 years old to 97 years old. The proportion of patients with DM was higher in controls than in PR cases. On the contrary, the percentages of patients with a history of gingival and periodontal diseases (ICD-9-CM: 523), acute or chronic PD (ICD-9-CM: 523.3–4), chronic PD (ICD-9-CM: 523.4), or PD (ICD-9-CM: 523.3–5) were consistently higher in PR cases than in controls. The numbers with proportions of patients treated with major PR-related medications, including DMARDs (i.e., hydroxychloroquine, methotrexate, and sulfasalazine), CSs, and NSAIDs, during the first year or the entirel follow-up period are listed in Table A in S1 Table. During the entire follow-up period, 96.4% of PR patients were treated with one more more PR-related medications, and 73.5% of PR patients used at least one DMARD.

**Table 1:** Demographic data and clinical characteristics among patients.

	Non-PR	PR	
	(*n* = 44,210)	(*n* = 4,421)	P-value
**Age, years** (mean ± SD)	46 ± 15	46 ± 15	1
<65years	39,120 (88.5)	3,912 (88.5)	
≥65years	5,090 (11.5)	509 (11.5)	
**Sex**			1
Female	31,130 (70.4)	3,113 (70.4)	
Male	13,080 (29.6)	1,308 (29.6)	
**Diabetes mellitus within 1 year before index date**	2,561 (5.8)	213 (4.8)	0.008
**Gingival and periodontal diseases** (ICD-9-CM: 523)	14,968 (33.9)	1,942 (43.9)	<0.001
**Acute or chronic periodontitis** (ICD-9-CM: 523.3–4)	9,988 (22.6)	1,370 (30.9)	<0.001
**Chronic periodontitis** (ICD-9-CM: 523.4)	2,344 (5.3)	311 (7.0)	<0.001
**Periodontitis** (ICD-9-CM: 523.3–5)	13,276 (30.0)	1,715 (38.8)	<0.001
**Time interval between the latest periodontitis-related visit and the index date**			<0.001
<3 months	1,072 (2.4)	199 (4.5)	
3–6 months	1,026 (2.3)	159 (3.6)	
6 months–1 year	1,825 (4.1)	241 (5.5)	
1–3 years	5,068 (11.5)	4.1)	
>3 years	4,285 (9.7)	493 (11.2)	
**Number of visits for periodontitis**			<0.001
Q1 (1–2)	4,153 (9.4)	375 (8.5)	
Q2 (3–4)	3,481 (7.9)	428 (9.7)	
Q3 (5–7)	2,990 (6.7)	427 (9.7)	
Q4 (≥8)	2,652 (6.0)	485 (11.0)	
**Cumulative cost of periodontitis-related visits (US$)**			<0.001
Q1 (1–54)	3,453 (7.8)	298 (6.7)	
Q2 (55–112)	3,418 (7.7)	384 (8.7)	
Q3 (113–202)	3,260 (7.4)	446 (10.1)	
Q4 (>202)	3,145 (7.1)	587 (13.3)	

Results are shown as number (%) unless specified otherwise.

Abbreviations: PR, palindromic rheumatism; SD, standard deviation; ICD-9-CM, International Classification of Diseases, Ninth revision, Clinical Modification; US$, United States dollars; Q, quartile.

As shown in Table 2, PD was associated with a higher risk of PR (OR, 1.50; 95% CI, 1.41–1.60), but DM was associated with a lower risk of PR (OR, 0.81; 95% CI, 0.70–0.94). After adjustment for the comorbidity of DM, the risk of PR was still higher in PD patients (OR, 1.50; 95% CI, 1.41–1.60).

**Table 2:** Crude and adjusted OR with 95% CI for correlation between variables and palindromic rheumatism risk using conditional logistic regression analyses.

Variable	Crude	Adjusted
Periodontitis (ICD-9-CM: 523.3–5)	1.50 (1.41–1.60)	1.51 (1.41–1.61)
Diabetes mellitus	0.81 (0.70–0.94)	0.79 (0.68–0.91)

Matched variables include age, sex and year of index date. Adjusted variable includes diabetes mellitus requiring anti-diabetic drugs.

Abbreviations: OR, odds ratio; CI, confidence interval; ICD-9-CM, International Classification of Diseases, Ninth Revision, Clinical Modification.

As shown in Table 3, the association between PD exposure history and PR risk was still statistically significant using different PD definitions based on the ICD-9-CM. However, the magnitude of this association mildly decreased on using ICD-9-CM code 523.4 as the PD definition.

**Table 3:** Sensitivity analyses comparing crude and adjusted ORs with 95% CIs for correlation between palindromic rheumatism risk and periodontitis using different definitions based on ICD-9-CM codes.

Periodontitis definition	Crude	Adjusted
Periodontitis (ICD-9-CM: 523.3–5)	1.50 (1.41–1.60)	1.51 (1.41–1.61)
Chronic periodontitis (ICD-9-CM: 523.4)	1.36 (1.20–1.54)	1.37 (1.21–1.55)
Acute or chronic periodontitis (ICD-9-CM: 523.3–4)	1.56 (1.46–1.67)	1.57 (1.46–1.68)
Gingival and periodontal diseases (ICD9-CM: 523)	1.56 (1.46–1.66)	1.56 (1.47–1.67)

Analyses were conducted using the conditional logistic regression model. Matched variables include age, sex and year of the index date. Adjusted variable includes diabetes mellitus requiring anti-diabetic drugs.

Abbreviations: OR, odds ratio; CI, confidence interval; ICD-9-CM, International Classification of Diseases, Ninth Revision, Clinical Modification.

As shown in Table 4, the magnitude of the association between PD and PR risk was greater if the time interval between the last PD-related visit and the index date was shorter, and when the number of PD-related visits or cumulative cost for PD was higher, suggesting a time- and dose-dependent association.

**Table 4:** Crude and adjusted OR with 95% CI for associations between palindromic rheumatism risk and history of periodontitis using conditional logistic regression analyses.

	Crude	Adjusted
**Time interval between the latest periodontitis-related visit and the index date**		
No periodontitis	1.00 (reference)	1.00 (reference)
<3 months	2.17 (1.85–2.53)	2.18 (1.86–2.55)
3–6 months	1.80 (1.51–2.13)	1.80 (1.52–2.14)
6 months–1 year	1.54 (1.33–1.77)	1.54 (1.34–1.78)
1–3 years	1.42 (1.30–1.56)	1.43 (1.30–1.57)
>3 years	1.34 (1.21–1.49)	1.35 (1.22–1.49)
**Number of visits for periodontitis**		
No periodontitis	1.00 (reference)	1.00 (reference)
Q1 (1–2)	1.05 (0.93–1.17)	1.05 (0.94–1.17)
Q2 (3–4)	1.43 (1.29–1.60)	1.44 (1.29–1.60)
Q3 (5–7)	1.68 (1.51–1.88)	1.69 (1.51–1.89)
Q4 (≥8)	2.19 (1.97–2.44)	2.20 (1.98–2.45)
**Cumulative cost of periodontitis-related visits, US$**		
No periodontitis	1.00 (reference)	1.00 (reference)
Q1 (1–54)	1.00 (0.88–1.13)	1.00 (0.88–1.13)
Q2 (55–112)	1.31 (1.17–1.47)	1.32 (1.18–1.47)
Q3 (113–202)	1.61 (1.44–1.79)	1.61 (1.45–1.80)
Q4 (>202)	2.25 (2.03–2.48)	2.25 (2.04–2.49)

Matched variables include age, sex and year of index date. Adjusted variable includes diabetes mellitus requiring anti-diabetic drugs.

Abbreviations: OR, odds ratio; CI, confidence interval; ICD9-CM, International Classification of Diseases, Ninth Revision, Clinical Modification; US$, United States dollars; Q, quartile.

As shown in Table 5, the association between PD and PR risk remained statistically significant among the subgroups stratified by age, sex, DM, or status of progression to RA, except in male patients or those aged ≥65 years using chronic PD as the PD definition. In addition, the association between PD and PR risk was significantly stronger among those aged <65 years than among those aged ≥65 years using ICD-9-CM: 523.3–4 or 523 as the PD definition (*p* for interaction = 0.019 and 0.023, respectively). Also, using ICD-9-CM code 523.4 as the PD definition, this association was significant among non-DM individuals but not among DM patients (*p* for interaction = 0.018). As shown in Table 5, although the association between PD and PR risk appears stronger among those who progressed to RA than that among nonprogressors, such difference did not reach statistical significance (*p* for interaction all > 0.05 in analyses using various definitions of PD).

**Table 5:** Multivariable analyses for the correlation of palindromic risk with a history of periodontitis using various definitions based on ICD-9-CM stratified by age, sex, or diabetes mellitus (DM).

	Periodontitis (ICD-9-CM: 523.3–5)		Chronic periodontitis (ICD-9-CM: 523.4)		Acute or chronic periodontitis (ICD-9-CM: 523.3–4)		Gingival and periodontal diseases (ICD-9-CM: 523)	
	OR (95% CI)	P for interaction	OR (95% CI)	P for interaction	OR (95% CI)	P for interaction	OR (95% CI)	P for interaction
**Age**		0.024		0.090		0.019		0.023
<65 years	1.55 (1.45–1.66)		1.43 (1.25–1.64)		1.62 (1.50–1.74)		1.61 (1.50–1.72)	
≥65 years	1.23 (1.02–1.48)		1.05 (0.75–1.46)		1.25 (1.02–1.52)		1.27 (1.05–1.53)	
**Sex**		0.154		0.113		0.094		0.158
Female	1.56 (1.44–1.68)		1.46 (1.26–1.69)		1.63 (1.50–1.77)		1.61 (1.49–1.74)	
Male	1.40 (1.24–1.58)		1.17 (0.93–1.48)		1.43 (1.26–1.62)		1.45 (1.29–1.64)	
**DM**		0.355		0.018		0.398		0.281
No	1.52 (1.42–1.63)		1.44 (1.27–1.64)		1.58 (1.47–1.70)		1.58 (1.48–1.69)	
Yes	1.65 (1.08–2.51)		1.06 (0.55–2.06)		1.79 (1.15–2.80)		1.51 (1.004–2.28)	
**Progression to RA**		0.179		0.134		0.547		0.449
No	1.48 (1.38–1.58)		1.41 (1.23–1.60)		1.55 (1.44–1.67)		1.54 (1.44–1.65)	
Yes	1.69 (1.41–2.02)		1.02 (0.68–1.52)		1.65 (1.36–2.00)		1.66 (1.39–1.98)	

Abbreviations: OR, odds ratio; CI, confidence interval; ICD-9-CM, International Classification of Diseases, Ninth revision, Clinical Modification; RA, rheumatoid arthritis.

A total of 569 PR patients developed RA and were issued a catastrophic illness certificate for RA. The time from index date to RA development was 1.03 ± 1.14 (mean ± SD) years. The remaining 3,852 PR patients did not progress to RA during a follow-up time of 3.17 ± 1.67 (mean ± SD) years. After exclusion of PR patients who progressed to RA, 3,852 PR patients and 38,520 non-PR controls matched for age, sex and the year of the index date were included in sensitivity analyses. As shown in Table B in S1 Table, the comparison of demographic and clinical characteristics between PR patients and non-PR controls is consistent with that in Table 1. The crude and adjusted ORs with 95% CIs for associations between PR risk and PD shown in Table C and D in S1 Table are also quite similar to the data shown in Table 2 and Table 3. Also, as shown in Table D in S1 Table, the crude and adjusted ORs with 95% CIs for associations between PD and PR risk using various PD definitions according to ICD-9-CM codes are consistent with the data shown in Table 3. As shown in Table E in S1 Table, a time- and dose-dependent relationship between PD and PR risk still existed. As shown in Table F in S1 Table, the association between PD and PR risk among subgroups stratified by age, sex or DM was also consistent with the data shown in Table 5.

We then used a case-control study design to examine the relationship between history of PD and PR treatment during the first year of follow-up among all PR patients (n = 4,421). As shown in Table G in S1 Table, history of PD was only associated with HCQ use, and such association was strongest for PD with the highest proxy measures of severity (Q4 of the number and costs for PR-related visits), suggesting a potential relationship between PD severity and PR severity in PR patients. Finally, we used a cohort study design to investigate the influence of the history of PD on the progression to RA among all PR patients (n = 4,421). As shown in Table H in S1 Table, no significant association was observed between the history of PD and the risk of progression to RA.

## Discussion

To the best of our knowledge, this study is the first of its kind to examine the association between a history of PD and PR risk. The main finding of this study was a statistically significant association between a history of PD and the risk of PR after adjustment for DM. This association was not sensitive to varying definitions of PD based on ICD-9-CM codes. Of note, the result was consistent after exclusion of PR patients who progressed to RA. Although misclassification of patients with PD based on ICD-9-CM codes may occur, the misclassification rate in cases and controls should be non-differential. Therefore, such misclassification bias due to miscoding of PD could only underestimate the association between PD exposure and PR risk [30]. Moreover, given the assumption that PD severity was positively correlated with the cumulative cost and number of visits for PD, we found a dose-response relationship between PD and PR risk. Also, our study revealed that the risk of PR was higher in PD patients with a shorter interval between their last PD-related visit and the index date. Taken together, these results suggest that PD is an environmental risk factor for the development of PR.

A possible explanation for our main finding is that a certain portion of PR patients with PD, whose disease later evolved to RA, had prior development of ACPA triggered by *P*. *gingivalis* expressed PAD (PPAD). However, the results were consistent after the exclusion of PR patients who developed RA after the index date. Also, the magnitude of the association between PD and PR was even higher than that of the association between PD and RA (1.51 vs. 1.16) [24]. Therefore, further studies are warranted to explore other possible mechanisms of our main finding, such as microbiome-triggered autoimmunity [31] or a shared genetic background.

A secondary finding of this study was that patients with DM requiring anti-diabetic therapy had a lower risk of PR. We also found a similar protective effect of treated DM on RA risk in our previous study [24]. Possible explanations included an immunosuppressive effect by hyperglycemia [32], or possible immunomodulatory effects of some anti-diabetic drugs [33–35].

In this study, the rate of progression of PR to RA in Taiwan (13% after a mean of 1.0 year) is lower than those in other countries [6,8–11]. Previous studies have shown that the presence of rheumatoid factor (RF) and/or ACPA is useful for predicting the progression of PR to RA [9,11,14,36–38]. Because the NHIRD lacked laboratory data, we cannot provide the prevalence of RF and ACPA of the study cohort. However, in our previous hospital-based study, the rates of positive ACPA and positive RF were only 13% and 14% in PR patients, which were also lower than those reported in PR patients in other countries [9,11,36–38]. In the present study, the rate of progression to RA was comparable to the progression rate of 15% after a mean of 1.4 years reported by our previous hospital-based study [14]. Therefore, it is reasonable to hypothesize that the low rate of progression to RA in Taiwanese PR patients in the study may be related to a low rate of positive RF and positive ACPA.

The use of population-based data in this study could minimize selection bias. However, several limitations should be addressed. First, the NHIRD lacked the information on potential confounding factors such as individual history of tobacco and alcohol use, socioeconomic status and family history of autoimmune diseases. Furthermore, laboratory data were not available, limiting further stratified analyses based on antibodies, such as rheumatoid factor and ACPA. Second, the major concern of the study is the misclassification of PR diagnosis. The accuracy of disease diagnoses based on administrative data is always of concern, though the BNHI has improved this aspect by regularly checking the original medical chart [26]. In addition, the rate of progression to RA was 13% after a mean of 1.0 year, which was comparable to the progression rate of 15% after a mean of 1.4 years reported by our previous hospital-based study [14]. Such consistency may suggest that the clinical characteristics of PR patients in the present study were at least partly comparable to those of PR patients in our previous study [14]. Third, we assumed that the severity of PD positively correlated with the number and costs of PD-related visits. However, such assumption cannot be proved, and the use of these measures to represent PD severity is of concern. Fourth, this is a case-control study, which has less power than a cohort study, to conclude a causal relationship. Finally, the study results cannot be generalized to populations outside Taiwan.

## Conclusion

This population-based, matched case-control study revealed an association between a history of PD and the risk of PR. The strength of this association increased with shorter lag time of the last PD-related visit or with greater severity of PD, using a cumulative visit number or cost of PD as a proxy for PD severity, suggesting a time- and dose-dependent relationship. Further clinical and basic studies are warranted to clarify the role of PD in PR development.

## Supporting information

S1 Table Supplementary Tables. (DOCX)

S1 Data Data of the PD group and the non-PD group. (SAV)
