# Supplementary material for: Association between periodontitis and the risk of palindromic rheumatism: A nationwide, population-based, case-control study
Source: PLoS One. 2017 Aug 3;12(8):e0182284. doi: 10.1371/journal.pone.0182284 (PMC5542542; doi:10.1371/journal.pone.0182284)
Supplement: S1 Table — (DOCX) [file pone.0182284.s001.docx]

**Association between Periodontitis and the Risk of Palindromic Rheumatism: A Nationwide, Population-Based, Case-Control Study**

**Authors:** Ching-Heng Lin^¶^, Der-Yuan Chen^¶^, Wen-Cheng Chao, Tsai-Ling Liao, Yi-Ming Chen, Hsin-Hua Chen^*^

**Supplemental materials**

| A. Numbers with proportions of PR patients treated with major PR-related medications during the entire follow-up period and during the first year (n = 4,421**)** | | | | | | |  |
| --- | --- | --- | --- | --- | --- | --- | --- |
|  | During the entire follow-up period | |  | During the first year | | |  |
| DMARDs | 3,248 (73.5) | |  | 3,027 (68.5) | | |  |
| Methotrexate | 666 (15.1) | |  | 509 (11.5) | | |  |
| Sulfasalazine | 1,317 (29.8) | |  | 1,105 (25.0) | | |  |
| **Hydroxychloroquine** | 2,944 (66.6) | |  | 2,718 (61.5) | | |  |
| Corticosteroids | 1,088 (40.7) | |  | 1,531 (34.6) | | |  |
| NSAIDs | 3,937 (89.1) | |  | 3,779 (85.5) | | |  |
| Any PR-related drug | | 4,263 (96.4) | | |  | 4,192 (94.8) | |

Abbreviations: PR, palindromic rheumatism; DMARDs, disease-modifying antirheumatic drugs; NSAIDs, nonsteroidal anti-inflammatory drugs.

B. Demographic data and clinical characteristics of subjects

|  | Non-PR (n = 38,520) |  | PR (n = 3,852) | *P*-value |
| --- | --- | --- | --- | --- |
| **Age, years** (mean ± SD) | 46 ± 15 |  | 46 ± 15 | 1.000 |
| Age group |  |  |  | 1.000 |
| <65 years | 33,870 (87.9) |  | 3,387 (87.9) |  |
| ≥65 years | 4,650 (12.1) |  | 465 (12.1) |  |
| **Sex** |  |  |  | 1.000 |
| Female | 26,770 (69.5) |  | 2,677 (69.5) |  |
| Male | 11,750 (30.5) |  | 1,175 (30.5) |  |
| **Diabetes mellitus within 1 year before index date** | 2,333 (6.1) |  | 201 (5.2) | 0.036 |
| **Gingival and periodontal diseases** (ICD-9-CM: 523) | 13,052 (33.9) |  | 1,693 (44.0) | <0.001 |
| **Acute or chronic periodontitis** (ICD-9-CM: 523.3–4) | 8,735 (22.7) |  | 1,197 (31.1) | <0.001 |
| **Chronic periodontitis** (ICD-9-CM: 523.4) | 1,955 (5.2) |  | 283 (7.4) | <0.001 |
| **Periodontitis** (ICD-9-CM: 523.3–5) | 11,528 (29.9) |  | 1,491 (38.7) | <0.001 |
| **Time interval between the last periodontitis-related visit and the index date** |  |  |  | <0.001 |
| <3 months | 901 (2.3) |  | 176 (4.6) |  |
| 3–6 months | 945 (2.5) |  | 140 (3.6) |  |
| 6 months–1 year | 1,581 (4.2) |  | 212 (5.5) |  |
| 1–3 years | 4,263 (11.1) |  | 531 (13.8) |  |
| >3 years | 3,838 (10.0) |  | 432 (11.2) |  |
| **Number of visits for periodontitis** |  |  |  | <0.001 |
| Q1 (≤2) | 3,674 (9.5) |  | 323 (8.4) |  |
| Q2 (3–4) | 2,930 (7.6) |  | 372 (9.7) |  |
| Q3 (5–7) | 2,569 (6.7) |  | 366 (9.5) |  |
| Q4 (≥8) | 2,355 (6.1) |  | 430 (11.2) |  |
| **Cumulative cost of periodontitis-related visits (US$)** |  |  |  | <0.001 |
| Q1 (0–54) | 3,051 (7.9) |  | 262 (6.8) |  |
| Q2 (55–112) | 2,945 (7.7) |  | 324 (8.4) |  |
| Q3 (113–207) | 2,842 (7.4) |  | 403 (10.5) |  |
| Q4 (>207) | 2,690 (7.0) |  | 502 (13.0) |  |

Results are shown as number (%) unless specified otherwise.

Abbreviations: PR, palindromic rheumatism; SD, standard deviation; ICD-9-CM, International Classification of Diseases, Ninth Revision, Clinical Modification; US$, United States dollars; Q, quartile.

C. Crude and adjusted OR with 95% CI for correlation between variables and palindromic rheumatism risk using conditional logistic regression analyses

| Variable | Crude |  | Adjusted |
| --- | --- | --- | --- |
| Periodontitis (ICD-9-CM: 523.3–5) | 1.50 (1.41–1.61) |  | 1.51 (1.41–1.62) |
| Diabetes mellitus | 0.84 (0.70–0.98) |  | 0.82 (0.71–0.96) |
| Matched variables include age, sex and year of index date. Adjusted variable includes diabetes mellitus requiring anti-diabetic drugs.  Abbreviations: OR, odds ratio; CI, confidence interval; ICD-9-CM, International Classification of Diseases, Ninth revision, Clinical Modification. | | | |

D. Sensitivity analyses comparing crude and adjusted ORs with 95% CIs for correlation between palindromic rheumatism risk and periodontitis using different definitions based on ICD-9-CM codes

| Periodontitis definition | Crude |  | Adjusted |
| --- | --- | --- | --- |
| Periodontitis (ICD-9-CM: 523.3–5) | 1.50 (1.41–1.61) |  | 1.51 (1.41–1.62) |
| Chronic periodontitis (ICD-9-CM: 523.4) | 1.46 (1.28–1.67) |  | 1.37 (1.29–1.67) |
| Acute or chronic periodontitis (ICD-9-CM: 523.3–4) | 1.56 (1.45–1.68) |  | 1.56 (1.45–1.68) |
| Gingival and periodontal diseases (ICD-9-CM: 523) | 1.56 (1.45–1.67) |  | 1.56 (1.46–1.67) |

Analyses were conducted using the conditional logistic regression model. Matched variables include age, sex and year of the index date. Adjusted variable includes diabetes mellitus requiring anti-diabetic drugs.

Abbreviations: OR, odds ratio; CI, confidence interval; ICD-9-CM, International Classification of Diseases, Ninth Revision, Clinical Modification.

E. Crude and adjusted OR with 95% CI for correlation between palindromic rheumatism risk and history of periodontitis using conditional logistic regression analyses

| Periodontitis status | Crude |  | Adjusted |
| --- | --- | --- | --- |
| **Time interval between the last periodontitis-related visit and the index date** |  |  |  |
| No periodontitis | 1.00 (reference) |  | 1.00 (reference) |
| <3 months | 2.17 (1.85–2.53) |  | 2.18 (1.86–2.55) |
| 3–6 months | 1.80 (1.51–2.13) |  | 1.80 (1.52–2.14) |
| 6 months–1 year | 1.54 (1.33–1.77) |  | 1.54 (1.34–1.78) |
| 1–3 years | 1.42 (1.30–1.56) |  | 1.43 (1.30–1.57) |
| >3 years | 1.34 (1.21–1.49) |  | 1.35 (1.22–1.49) |
| **Number of visits for periodontitis** |  |  |  |
| No periodontitis | 1.00 (reference) |  | 1.00 (reference) |
| Q1 (≤2) | 1.05 (0.93–1.17) |  | 1.05 (0.94–1.17) |
| Q2 (3–4) | 1.43 (1.29–1.60) |  | 1.44 (1.29–1.60) |
| Q3 (5–7) | 1.68 (1.51–1.88) |  | 1.69 (1.51–1.89) |
| Q4 (≥8) | 2.19 (1.97–2.44) |  | 2.20 (1.98–2.45) |
| **Cumulative cost of periodontitis-related visits, US$** |  |  |  |
| No periodontitis | 1.00 (reference) |  | 1.00 (reference) |
| Q1 (0–54) | 1.00 (0.88–1.13) |  | 1.00 (0.88–1.13) |
| Q2 (55–112) | 1.31 (1.17–1.47) |  | 1.32 (1.18–1.47) |
| Q3 (113–202) | 1.61 (1.44–1.79) |  | 1.61 (1.45–1.80) |
| Q4 (>202) | 2.25 (2.03–2.48) |  | 2.25 (2.04–2.49) |

Matched variables include age, sex and year of index date. Adjusted variable includes diabetes mellitus requiring anti-diabetic drugs.

Abbreviations: OR, odds ratio; CI, confidence interval; ICD-9-CM, International Classification of Diseases, Ninth Revision, Clinical Modification; US$, United States dollars; Q, quartile..

Table F Multivariable analyses for the correlation of palindromic risk with a history of periodontitis using various definitions based on ICD--CM stratified by age, sex or diabetes mellitus (DM)

|  | Periodontitis (ICD-9-CM: 523.3–5) | |  | Chronic periodontitis (ICD-9-CM: 523.4) | |  | Acute or chronic periodontitis (ICD-9-CM: 523.3–4) | |  | Gingival and periodontal diseases (ICD-9-CM: 523) | |
| --- | --- | --- | --- | --- | --- | --- | --- | --- | --- | --- | --- |
|  | OR (95% CI) | P for interaction |  | OR (95% CI) | P for interaction |  | OR (95% CI) | P for interaction |  | OR (95% CI) | P for interaction |
| **Age** |  | 0.024 |  |  | 0.090 |  |  | 0.019 |  |  | 0.023 |
| <65 years | 1.55 (1.45–1.66) |  |  | 1.43 (1.25–1.64) |  |  | 1.62 (1.50–1.74) |  |  | 1.61 (1.50–1.72) |  |
| ≥65 years | 1.23 (1.02–1.48) |  |  | 1.05 (0.75–1.46) |  |  | 1.25 (1.02–1.52) |  |  | 1.27 (1.05–1.53) |  |
| **Sex** |  | 0.154 |  |  | 0.113 |  |  | 0.094 |  |  | 0.158 |
| Female | 1.56 (1.44–1.68) |  |  | 1.46 (1.26–1.69) |  |  | 1.63 (1.50–1.77) |  |  | 1.61 (1.49–1.74) |  |
| Male | 1.40 (1.24–1.58) |  |  | 1.17 (0.93–1.48) |  |  | 1.43 (1.26–1.62) |  |  | 1.45 (1.29–1.64) |  |
| **DM** |  | 0.355 |  |  | 0.018 |  |  | 0.398 |  |  | 0.281 |
| No | 1.52 (1.42–1.63) |  |  | 1.44 (1.27–1.64) |  |  | 1.58 (1.47–1.70) |  |  | 1.58 (1.48–1.69) |  |
| Yes | 1.65 (1.08–2.51) |  |  | 1.06 (0.55–2.06) |  |  | 1.79 (1.15–2.80) |  |  | 1.51 (1.004–2.28) |  |

Abbreviations: OR, odds ratio; CI, confidence interval; ICD-9-CM, International Classification of Diseases, Ninth Revision, Clinical Modification.

Table G. Crude and multivariable adjusted* analyses for the associations between PD and PR-related medication use during the first year of follow-up in all PR patients (n = 4,421), shown as odds ratios with 95% confidence intervals.

|  | Methotrexate | |  | Sulfasalazine | |  | Hydroxychloroquine | |  | Corticosteroids | |
| --- | --- | --- | --- | --- | --- | --- | --- | --- | --- | --- | --- |
|  | Crude | Adjusted |  | Crude | Adjusted |  | Crude | Adjusted |  | Crude | Adjusted |
| **Various periodontitis definition** |  |  |  |  |  |  |  |  |  |  |  |
| No periodontitis | 1.00 (reference) | 1.00 (reference) |  | 1.00 (reference) | 1.00 (reference) |  | 1.00 (reference) | 1.00 (reference) |  | 1.00 (reference) | 1.00 (reference) |
| Gingival and periodontal diseases | 0.97 (0.80–1.17) | 0.99 (0.82–1.19) |  | 0.97 (0.85–1.12) | 1.00 (0.87–1.15) |  | 1.16 (1.02–1.31) | 1.20 (1.06–1.36) |  | 1.06 (0.94–1.20) | 1.08 (0.95–1.22) |
| Acute or chronic periodontitis | 0.87 (0.71–1.07) | 0.89 (0.73–1.10) |  | 0.95 (0.82–1.11) | 0.99 (0.85–1.14) |  | 1.20 (1.05–1.37) | 1.24 (1.09–1.42) |  | 1.04 (0.91–1.19) | 1.05 (0.92–1.21) |
| Chronic periodontitis | 0.78 (0.53–1.15) | 0.83 (0.56–1.23) |  | 0.81 (0.62–1.08) | 0.89 (0.67–1.18) |  | 0.97 (0.77–1.23) | 1.07 (0.84–1.36) |  | 0.93 (0.73–1.19) | 0.97 (0.76–1.25) |
| Periodontitis | 0.91 (0.75–1.10) | 0.94 (0.77–1.13) |  | 0.96 (0.83–1.10) | 1.00 (0.87–1.16) |  | 1.17 (1.03–1.33) | 1.24 (1.09–1.41) |  | 1.06 (0.93–1.20) | 1.08 (0.95–1.23) |
| **Time interval between the latest periodontitis-related visit and the index date** |  |  |  |  |  |  |  |  |  |  |  |
| No periodontitis | 1.00 (reference) | 1.00 (reference) |  | 1.00 (reference) | 1.00 (reference) |  | 1.00 (reference) | 1.00 (reference) |  | 1.00 (reference) | 1.00 (reference) |
| <3 months | 0.65 (0.38–1.09) | 0.68 (0.40–1.14) |  | 0.86 (0.61–1.22) | 0.92 (0.65–1.30) |  | 1.10 (0.82–1.48) | 1.19 (0.88–1.61) |  | 0.87 (0.64–1.19) | 0.90 (0.66–1.23) |
| 3–6 months | 0.83 (0.49–1.41) | 0.86 (0.51–1.46) |  | 0.74 (0.50–1.11) | 0.79 (0.53–1.17) |  | 0.99 (0.71–1.37) | 1.06 (0.76–1.48) |  | 0.99 (0.71–1.39) | 1.02 (0.73–1.43) |
| 6 months–1 year | 1.05 (0.71–1.57) | 1.09 (0.73–1.62) |  | 0.87 (0.64–1.19) | 0.91 (0.67–1.25) |  | 1.02 (0.78–1.34) | 1.08 (0.82–1.41) |  | 1.11 (0.84–1.46) | 1.14 (0.86–1.49) |
| 1–3 years | 0.89 (0.68–1.18) | 0.92 (0.70–1.22) |  | 1.04 (0.85–1.27) | 1.09 (0.89–1.33) |  | 1.28 (1.07–1.54) | 1.37 (1.14–1.65) |  | 1.05 (0.87–1.26) | 1.07 (0.89–1.29) |
| >3 years | 0.99 (0.73–1.33) | 1.01 (0.75–1.36) |  | 1.01 (0.81–1.26) | 1.05 (0.84–1.31) |  | 1.21 (0.99–1.48) | 1.27 (1.04–1.56) |  | 1.15 (0.94–1.40) | 1.16 (0.95–1.42) |
| **Number of visits for periodontitis** |  |  |  |  |  |  |  |  |  |  |  |
| No periodontitis | 1.00 (reference) | 1.00 (reference) |  | 1.00 (reference) | 1.00 (reference) |  | 1.00 (reference) | 1.00 (reference) |  | 1.00 (reference) | 1.00 (reference) |
| Q1 (1–3) | 0.85 (0.60–1.20) | 0.80 (0.60–1.08) |  | 0.89 (0.72–1.10) | 0.90 (0.73–1.11) |  | 0.93 (0.77–1.11) | 0.95 (0.79–1.14) |  | 1.16 (0.96–1.40) | 1.17 (0.97–1.41) |
| Q2 (4–5) | 1.02 (0.73–1.44) | 0.87 (0.61–1.23) |  | 0.80 (0.62–1.03) | 0.83 (0.64–1.07) |  | 1.39 (1.11–1.75) | 1.45 (1.15–1.82) |  | 0.86 (0.68–1.08) | 0.87 (0.69–1.09) |
| Q3 (6–8) | 1.03 (0.75–1.42) | 1.07 (0.76–1.51) |  | 1.07 (0.83–1.38) | 1.14 (0.88–1.47) |  | 1.18 (0.93–1.48) | 1.27 (1.003–1.61) |  | 1.26 (1.01–1.59) | 1.31 (1.04–1.65) |
| Q4 (>8) | 0.85 (0.60–1.20) | 1.11 (0.80–1.54) |  | 1.13 (0.89–1.44) | 1.27 (0.99–1.61) |  | 1.41 (1.13–1.77) | 1.61 (1.28–2.02) |  | 0.95 (0.76–1.19) | 1.00 (0.80–1.26) |
|  |  |  |  |  |  |  |  |  |  |  |  |
| **Cumulative cost of periodontitis-related visits, US$** |  |  |  |  |  |  |  |  |  |  |  |
| No periodontitis | 1.00 (reference) | 1.00 (reference) |  | 1.00 (reference) | 1.00 (reference) |  | 1.00 (reference) | 1.00 (reference) |  | 1.00 (reference) | 1.00 (reference) |
| Q1 (1–71) | 0.80 (0.57–1.12) | 0.80 (0.57–1.13) |  | 0.78 (0.61–0.99) | 0.79 (0.61–1.01) |  | 0.87 (0.71–1.07) | 0.89 (0.72–1.10) |  | 1.08 (0.87–1.33) | 1.09 (0.88–1.35) |
| Q2 (72–141) | 0.89 (0.64–1.24) | 0.92 (0.66–1.27) |  | 0.91 (0.72–1.16) | 0.95 (0.74–1.21) |  | 1.27 (1.03–1.58) | 1.35 (1.09–1.68) |  | 0.99 (0.80–1.23) | 1.01 (0.81–1.25) |
| Q3 (142–249) | 0.90 (0.65–1.24) | 0.92 (0.66–1.28) |  | 1.07 (0.85–1.34) | 1.11 (0.88–1.41) |  | 1.16 (0.94–1.44) | 1.23 (0.99–1.52) |  | 1.11 (0.90–1.38) | 1.14 (0.92–1.41) |
| Q4 (>249) | 1.04 (0.77–1.42) | 1.11 (0.81–1.52) |  | 1.09 (0.87–1.38) | 1.21 (0.96–1.52) |  | 1.48 (1.19–1.84) | 1.67 (1.33–2.08) |  | 1.05 (0.85–1.30) | 1.10 (0.88–1.36) |

*Adjusted variables included age, gender, and diabetes mellitus. Abbreviations: US$, United States dollars; Q, quartile.

Table H. The association between periodontitis status and the risk of progression to RA among all PR patients (n = 4,421) examined by crude and multivariable adjusted* Cox regression analysis shown as hazard ratios with 95% confidence intervals.

| Periodontitis status | Crude |  | Adjusted* |
| --- | --- | --- | --- |
| **Various periodontitis definitions** |  |  |  |
| No periodontitis | 1.00 (reference) |  | 1.00 (reference) |
| Gingival and periodontal diseases (ICD9: 523) | 1.08 (0.92–1.28) |  | 1.08 (0.92–1.28) |
| Acute or chronic periodontitis (ICD9: 523.3–4) | 1.07 (0.90–1.28) |  | 1.07 (0.90–1.28) |
| Chronic periodontitis (ICD9: 523.4) | 0.73 (0.50–1.07) |  | 0.73 (0.50–1.06) |
| Periodontitis (ICD9: 523.3–5) | 1.12 (0.95–1.33) |  | 1.13 (0.95–1.33) |
| **Time interval between the last periodontitis-related visit and the index date** |  |  |  |
| No periodontitis | 1.00 (reference) |  | 1.00 (reference) |
| <3 months | 0.95 (0.62–1.45) |  | 0.96 (0.63–1.46) |
| 3–6 months | 1.02 (0.64–1.61) |  | 1.03 (0.65–1.64) |
| 6 months–1 year | 0.98 (0.67–1.44) |  | 0.99 (0.68–1.45) |
| 1–3 years | 1.25 (0.99–1.57) |  | 1.24 (0.98–1.56) |
| >3 years | 1.14 (0.87–1.50) |  | 1.16 (0.88–1.52) |
| **Number of visits for periodontitis** |  |  |  |
| No periodontitis | 1.00 (reference) |  | 1.00 (reference) |
| Q1 (1–3) | 1.05 (0.82–1.35) |  | 1.07 (0.84–1.38) |
| Q2 (4–5) | 1.18 (0.89–1.57) |  | 1.18 (0.89–1.56) |
| Q3 (6–8) | 1.28 (0.95–1.72) |  | 1.28 (0.95–1.72) |
| Q4 (>8) | 1.02 (0.74–1.40) |  | 1.02 (0.74–1.41) |
| **Cumulative cost of periodontitis-related visits, US$** |  |  |  |
| No periodontitis |  |  |  |
| Q1 (1–71) | 1.19 (0.91–1.55) |  | 1.20 (0.92–1.57) |
| Q2 (72–141) | 1.05 (0.79–1.40) |  | 1.06 (0.79–1.40) |
| Q3 (142–249) | 1.21 (0.92–1.60) |  | 1.21 (0.92–1.60) |
| Q4 (>249) | 1.03 (0.75–1.40) |  | 1.03 (0.76–1.40) |

*Adjusted for age, gender, and diabetes mellitus. Abbreviations: US$, United States dollars; Q, quartile.
